# A Meta-analysis of Outcome Studies of Autistic Adults: Quantifying Effect Size, Quality, and Meta-regression

**DOI:** 10.1007/s10803-020-04763-2

**Published:** 2020-11-17

**Authors:** David Mason, Simone J. Capp, Gavin R. Stewart, Matthew J. Kempton, Karen Glaser, Patricia Howlin, Francesca Happé

**Affiliations:** 1 Social, Genetic, and Developmental Psychiatry Centre, Institute of Psychiatry, Psychology and Neuroscience, King’s College London, De Crespigny Park, Denmark Hill, London, SE5 8AF UK; 2 Department of Psychosis Studies, Institute of Psychiatry, Psychology and Neuroscience, King’s College London, 16 De Crespigny Park, Camberwell, London, SE5 8AF UK; 3 Department of Global Health & Science Medicine, King’s College London, Bush House North East Wing, 30 Aldwych, London, WC2B 4BG UK; 4 Department of Psychology, Institute of Psychiatry, Psychology and Neuroscience, King’s College London, 16 De Crespigny Park, Camberwell, London, SE5 8AF UK

**Keywords:** Autism spectrum disorder, Outcome, Functioning, Meta-analysis

## Abstract

Longitudinal studies have generally reported poor outcomes in adulthood for the majority of individuals (c.50–60%) with autism. Several factors putatively predict outcome (e.g. IQ), but findings remain mixed. This paper presents an updated meta-analysis of autism outcome studies and extends previous findings with additional analyses (including meta-regression). A total of 4088 records was screened and 18 studies, involving 1199 individuals, were included in the quantitative analysis. Estimated percentages indicated that 20.0% of participants were rated as having a good outcome, 26.6% a fair outcome, and 49.3% a poor outcome. Meta-regression indicated that lower IQ in adulthood was predictive of poor outcome; other meta-regression models did not survive correction for multiple comparisons. Overall, outcomes for autistic people are on average poor, and higher IQ appears to be protective against having a poor outcome. The limitations of current constructs of outcome are discussed.

**Electronic supplementary material:**

The online version of this article (10.1007/s10803-020-04763-2) contains supplementary material, which is available to authorized users.

## Introduction

Autism spectrum disorder (ASD)^1^ is currently conceptualised as a life-long neurodevelopmental condition, with impairments in social interaction, communication (both verbal and non-verbal), repetitive behaviours and/or restricted interests (American Psychological Association 2013). Although much existing research on autism focuses on children, recent studies have begun to focus more on outcomes in adulthood (Farley et al. 2018; Lounds Taylor 2017). The first longitudinal follow-up studies of autism defined a “good” adult outcome as “leading a normal, or near normal, social life” and “functioning satisfactorily at school or work”; “fair” was defined as an individual who made social and educational progress in spite of abnormal behaviour, or interpersonal relationships; “poor” was defined as an individual who was unable to lead an independent life, but where there was “some potential for social progress”; and “very poor” was defined as an individual unable to lead “any kind of independent existence” (Rutter et al. 1967, p. 1185). These definitions of outcome are provided for historical context, and are no longer used in research into outcomes for autistic people, as will be discussed below. Using similar criteria, subsequent follow-up studies concluded that between 50 to 60% of autistic people had poor, or very poor, outcomes (Gillberg and Steffenburg 1987; DeMyer et al. 1973; Larsen and Mouridsen 1997; Lotter 1974; Rutter et al. 1967). Thus, many participants were highly dependent on others, had limited friendships or relationships, and had poor prospects for education and employment. But, definitions of traditional outcomes at this time were inconsistent, thus confounding comparisons of outcome between studies (Henninger and Taylor 2013; Ruble and Dalrymple 1996). Subsequently, concepts of what constitutes a good outcome or “normal” or “near normal” social life have been criticised as being subjective (e.g. requiring interpretation by the researcher; Henninger and Taylor 2013). Other criticisms of traditional outcomes include: complaints that they are based on “normative” criteria that are not necessarily important for people with autism (Bishop-Fitzpatrick et al. 2016); or are too restricted and do not cover life domains such as communication, pain, well-being and independence (McConachie et al. 2018); or for overlooking personal factors that may be important for outcome (such as quality of life, Ruble and Dalrymple 1996; or within-individual change over time, Georgiades and Kasari 2018).

Henninger and Taylor (2013) note that the early 2000s marked a period of more empirically grounded, and standardised, measures of outcome, albeit still focused on participants’ capacity for sustaining an independent life. For instance, Howlin et al. (2004) measured outcome across three domains: employment, independent living, and friendships/romantic relationships, with hierarchical ratings reflecting different levels of attainment in these areas. Several other studies have used a similar framework to that of Howlin et al. (2004) (e.g. Eaves and Ho 2008; Farley et al. 2009, 2018; Howlin et al. 2013). Others suggest an alternative framework. For example, Billstedt et al. (2005), set age as a consideration in determining whether friendships *or* employment should be an indicator of outcome (if the participant was aged 23 years or older, living status was taken as an indicator of outcome; otherwise friendships/relationships were used as an outcome indicator). These authors also made a distinction between a fair (being in employment/education *or* living independently/having friendships) and a restricted but acceptable outcome (meeting neither of the former criteria but being in a context that is accepting of the individual).

Despite these and other modifications to outcome ratings, most recent studies still indicate that a large proportion of autistic adults have a poor or very poor outcome (Howlin and Magiati 2017; Magiati et al. 2014). Nevertheless, some have reported more promising outcomes. For instance, Farley et al. (2009) reported fair or better outcomes for 82% of their sample, likewise Kobayashi et al. (1992) described approximately 55% of participants as having fair or better outcomes (indicating more independence in employment, social relationships, and living independently). Farley and colleagues suggested that the higher rates of positive outcomes in their cohort may have been due to the fact that most participants (93%) were members of The Church of Jesus Christ of Latter Day Saints (LDS; note, membership of this Church was not a selection criterion) in which “Inclusion of individuals with disabilities is a strong cultural value” (Farley et al. 2009, p. 116). Kobayashi and colleagues also noted that many participants (82%) were attending therapeutic camps that specifically aimed to include autistic people in the social environment. Thus, although most studies report that outcomes for autistic people are often poor, it may be that contextual or social factors are related to better outcomes.

Overall, existing data illustrate that outcomes for autistic adults, as currently measured, are often poor in terms of employment, relationships, and independence, and recent research has begun to try to identify participant characteristics that may predict outcome. The predictive variables most frequently studied include IQ, autism symptom severity, and language (such as no speech, single word use, functional speech, or more fluent speech, Howlin et al. 2013; or communicative phrase speech by a certain age, Farley et al. 2009). When interpreting findings from this research it is essential to bear in mind that many studies use different measures for these predictors. Moreover, given the heterogeneity of samples, year of study publication, and age of each sample, it is difficult to identify the exact influence of these predictors on later adult outcomes. Finally, it is important to consider that studies published in the same year may report on cohorts of autistic people initially assessed at very different time points (for example, Cederlund et al. (2008) collected their childhood data in the early 2000s, whereas Eaves and Ho (2008) collected their childhood data in the 1980s). This has implications for comparing outcomes from contemporaneously published studies, as the participants in each sample may be at different life stages.

Several follow-up studies suggest that IQ remains relatively stable over time at the group level (Billstedt et al. 2005; Anderson D.K. et al. 2014; Cederlund et al. 2008; Howlin et al. 2013). In many other studies, higher childhood IQ is associated with more positive outcomes. Both Eaves and Ho (2008) and Kobayashi et al. (1992) report significant positive correlations between childhood IQ and adult outcome scores (with correlation coefficients ranging between 0.39 and 0.58). Gillberg and Steffenburg (1987) reported that an IQ of more than 50 distinguished those with fair or better outcomes from those with poor or very poor outcomes; Howlin et al. (2004) found that those with a poor or very poor outcome had a performance IQ (PIQ) below 70 *and* verbal IQ (VIQ) below 50. Having a higher IQ in adulthood is also associated with better outcome (Cederlund et al. 2008; Eaves and Ho 2008; Howlin et al. 2013; Jónsdóttir et al. 2018). Eaves and Ho (2008), Howlin et al., (2013) and Farley et al. (2009) report significant correlations (ranging from 0.40 to 0.65) between measures of IQ and outcome. Nevertheless, higher IQ alone does not guarantee a good prognosis; several studies report that participants with a higher IQ do not necessarily have better outcomes. Howlin et al. (2004) reported that individuals with IQ in the range 70–99 and those with an IQ ≥100 did not significantly differ in terms of outcome. Likewise Billstedt et al. (2005) reported that no-one in their study of 108 participants had a good outcomes despite 10% of their sample having an IQ greater than 70. Moreover, several studies have reported that at the participant level, IQ trajectories are not necessarily stable across time points (Billstedt et al. 2005; Cederlund et al. 2008; Farley et al. 2009; Howlin et al. 2004). Thus, IQ may be protective against the poorest outcome profile, but does not guarantee a good outcome (Pickles et al. 2020). The mixed results may be explained by individual differences in other factors, such as level of social support (Farley et al. 2009; Kobayashi et al. 1992), or severity of autism symptoms.

Autism severity in childhood has an even more complex relationship with outcome. Pickles et al. (2020) identified four classes of outcome based on a longitudinal cohort study (mean age at recruitment 3.3 years and 26.1 at follow-up). Although the class with the best outcome profile had uniformly high IQ, the authors also identified a class with high IQ but with poorer behavioural and affective outcomes. Howlin et al. (2013) found that retrospective information on early autism severity was a stronger predictor of outcome than either childhood language ability or childhood IQ. Helles et al. (2017) and Jónsdóttir et al. (2018) reported that autism severity (measured at mean age 29.9 and 6.0 years respectively) negatively correlated with outcome. Chamak and Bonniau (2016) also reported that “high functioning” autism/Asperger's was exclusively associated with good outcome, and those who had “moderate autism” or “severe autism” were more likely to have poor or very poor outcome. However, this study did not explicitly state how these three categories of autism were derived, and no assessment of cognitive ability was made. Eaves and Ho (2008) reported significant correlations between both VIQ and autism severity, but only VIQ accounted for a significant proportion of the variance in outcome; adding autism severity did not explain significantly more variance. It is also worth noting that autism severity seems to vary across the lifespan and may interact with IQ. Although Seltzer et al. (2003) observed a steady decrease across a range of autism symptom domains across the lifespan (i.e. from age 10 to 53 years), Anderson D.K. et al. (2014) reported that changes in autism symptomatology were greater for those with higher IQ in childhood.

Finally, early language development may also play a role in influencing outcome, although definitions of language vary considerably (from single words to communicative phrase speech, to expressive *and* receptive language). Several studies have reported that the presence of language (compared to no language) before the age of 5 or 6 years (Howlin et al. 2004; Sevaslidou et al. 2019) was associated with better outcome. Gillespie-Lynch et al. (2012) found that outcome was correlated with language ability (measured by expressive and receptive scores on the Reynell Scales of Language Ability Edwards et al. 1999) and with the extent of change in language ability between 4 and 18 years. In adulthood, poorer language ability (as measured by the Autism Diagnostic Interview-Revised; no phrase speech; phrase speech; or functional speech) was associated with a poorer outcome (Howlin et al. 2013).

A recent meta-analysis by Steinhausen et al. (2016) of 15 adult outcome studies reported pooled estimates for percentages with good, fair, and poor composite outcomes as 19.7%, 31.1%, and 47.7% respectively. The review also identified characteristics that were significantly related to outcome, principally autism diagnosis type and mean age at follow-up. The Steinhausen review is relatively recent, and is valuable in summarising data on the overall outcomes for autistic people. It also explores the putative relationships between outcome and diagnostic ‘type’ (individuals diagnosed with childhood autism had significantly poorer outcomes compared to those with broader diagnoses of autism spectrum disorder or Asperger Syndrome for fair and poor outcome), and potential age effects on outcome (good outcomes are more prevalent in early adulthood aged samples—those aged between 20 and 29 at follow-up). However, an updated review and meta-analysis of composite outcome studies is timely for four reasons.

First, several outcome studies have been published since the last meta-analysis by Steinhausen et al. (2016) (e.g. Farley et al. 2018; Helles et al. 2017; Sevaslidou et al. 2019; Pickles et al. 2020). Their inclusion is important in estimating outcomes for autistic people. For instance, Farley et al. (2018) report on a larger sample than their previous follow-up; they also included participants who were not diagnosed with DSM-III but were subsequently deemed to meet DSM-IV criteria. The resulting outcome data were less positive than previously reported (Farley et al. 2009). Further, Sevaslidou et al. (2019), having stratified their sample by gender, found that outcome was significantly poorer for males (although these findings are tentative as there were only 9 females in the sample). Second, the Steinhausen et al. review (2016) did not assess the quality, or risk of bias, in the studies included. This is important, as conclusions drawn from the literature should be considered in relation to the methodological quality of the research base. In addition, the authors did not conduct sensitivity analyses to determine the robustness of their findings (Patsopoulos et al. 2008). This is necessary to assess whether one or more studies is having an undue effect on the estimated effect. Finally, given the uncertainties about the relationships between IQ, autism severity, language, and composite outcome score, a meta-regression analysis would be an important methodological advance to address the relative roles of these variables in predicting outcome. Thus, the present study sought to replicate and extend the meta-analysis of Steinhausen et al. (2016) in four important ways: (i) produce an up-to-date, systematic review of outcome studies; (ii) replicate the sub-group analyses conducted by Steinhausen and colleagues; (iii) conduct sensitivity analyses to investigate how robust the estimates of outcome are; and (iv) investigate the predictive role of language, autism severity and IQ in a meta-regression model. This study focuses on the same conceptualisation of outcome (objective indicators based on employment, social relationships, and independent living). This is primarily because of the high heterogeneity reported in the previous meta-analysis of outcome studies, and because measures of outcome vary between study. Thus, it was deemed more methodologically rigorous to focus on this specific assessment of outcome.

## Methods

The Preferred Reporting Items for Systematic Reviews and Meta-Analyses (PRISMA) guidelines were used throughout the review (Moher et al. 2009).

### Review Criteria

All of the studies included in the meta-analysis of Steinhausen et al. (2016) were included in the present review. Relevant databases were searched from 2000 onwards, to ensure the present review was comprehensive.

Studies were eligible for inclusion if they:

- Were published from 2000 onwards
- Were peer-reviewed
- Contained a sample of autistic participants
- Were quantitative studies that reported primary data (cross-sectional studies were eligible if they reported the outcomes detailed below)
- Reported data about the three areas of outcome typically used in outcome studies (employment, independent living, relationships)
- Were studies that reported outcomes for adults (i.e. those samples with a mean age of 18+)
- Were published in English.

Studies were excluded if they failed to meet one or more inclusion criteria or were based on case studies. Qualitative studies, book chapters, Masters or PhD theses, other reviews, editorials etc. were also excluded.

### Search Strategy

The first author designed the search strategy based on previous reviews of outcome studies (Kirby et al. 2016; Howlin and Magiati 2017; Magiati et al. 2014; Steinhausen et al. 2016). The following search terms were used: autism spectrum disorder, Asperger's syndrome, adult, adolescent, teenager, outcome, longitudinal, trajectory, prognosis, and predict. Subject headings and MeSH terms were used for each database. Each search term without an associated subject or MeSH term was truncated and a wildcard was used for each term to increase the number of records returned (e.g. 'prognos*’ would return prognosis, prognostic etc.). Searches for terms were restricted to title and abstract level to prevent the return of too many false negatives (Magiati et al. 2014; Steinhausen et al. 2016). An example search strategy for the PsychINFO database can be found in Supplementary Material 1.

**Fig. 1:**
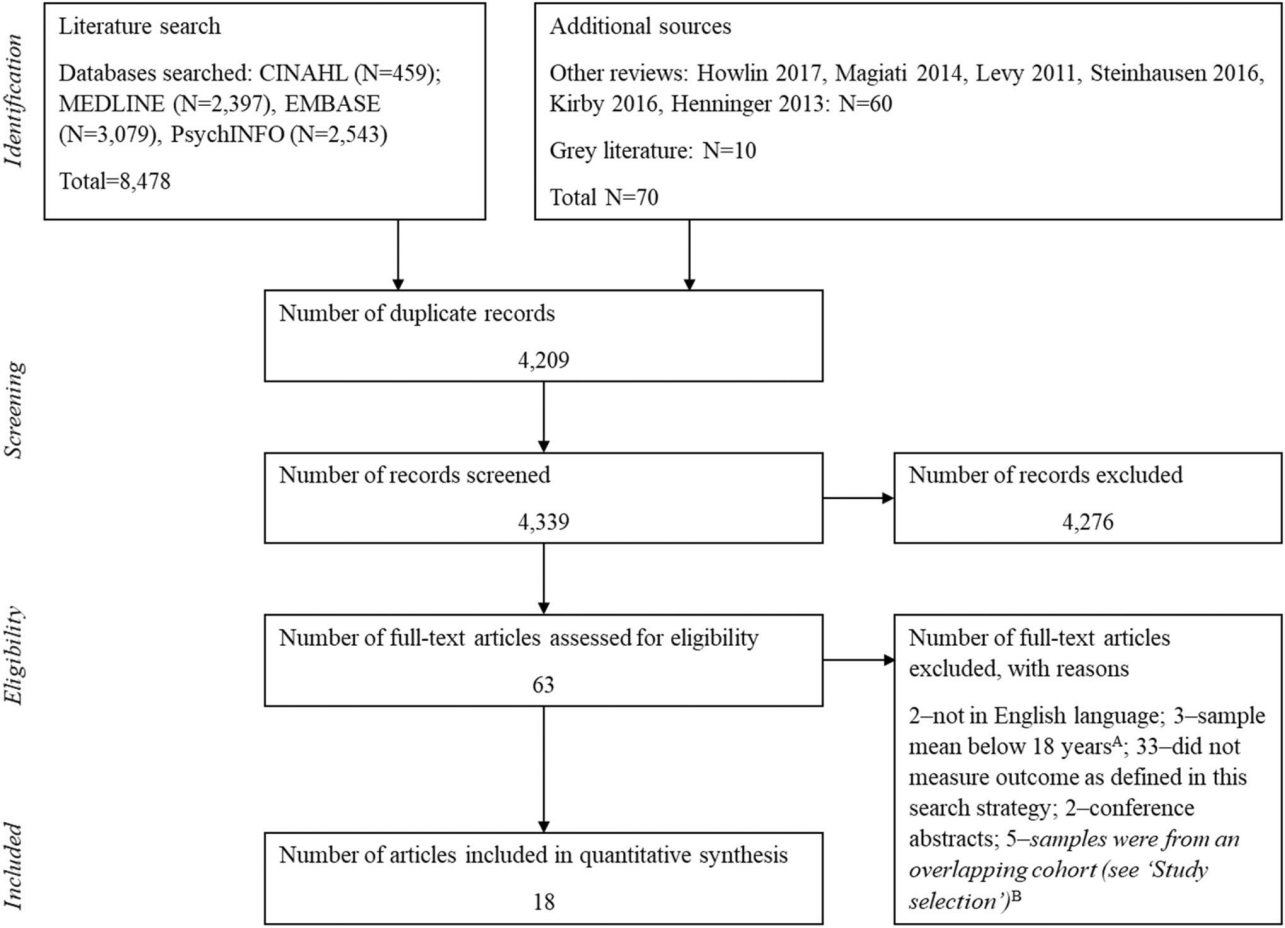
PRISMA diagram for the systematic review process. **a** These studies were included in the Steinhausen et al. (2016) review: DeMyer et al. (1973), Lotter (1974), Rutter et al. (1967). **b** Three of these were included in the Steinhausen et al. (2016) review: Farley et al. (2009), Howlin et al. (2004, 2013)

The following databases were searched on the 27th of October 2019: CINAHL, MEDLINE, Embase, PsycINFO. All searches were updated on the 30th of April 2020. To ensure maximal inclusion of potentially relevant studies, the reference lists of three reviews (Kirby et al. 2016; Magiati et al. 2014; Steinhausen et al. 2016) and the reference lists of all included studies were screened. Endnote referencing software was used to store, de-duplicate, and screen all included studies. See Fig. 1 for the PRISMA flowchart of the final database search.

### Defining Outcome

Outcome has been operationalised differently across studies (for a detailed discussion see Henninger and Taylor 2013). As noted in the introduction, Rutter et al. (1967) defined four outcome groups: good/normal, fair, poor, and very poor. In subsequent studies (e.g. Howlin et al. 2004), assessments of outcome have tended to be based on composite measures including occupation, friendships, and independent living, Overall ratings are generally summarised as very good /good, fair, poor, and/or very poor outcomes. Any studies using these, or a similar operationalisation of outcome, were included as the measure of interest for the review (see Supplementary Material 2 for further details). There are two important points to note. First, this analysis used the percentage in each outcome category as originally reported in each study. There is only one case where the research team used the Howlin criteria to derive outcome scores, as domain specific data only (for employment, living status, and friendships/relationships; see Supplementary Material 2) were provided, by personal communication (Pickles et al. 2020). Second, we collapsed the outcome categories into three groups for statistical reasons, details can be found in ‘Summary measures’.

### Study Selection Process

After deleting duplicate records the first author screened all titles and abstracts against the selection criteria. A second reviewer, GRS, checked a randomly selected 10% of the abstracts against the selection criteria to investigate agreement (98.5%). The first author then completed a full-text read-through of all papers included in the first screen against the selection criteria.

A standardised data capture form was used to collect the following data from all included papers: year of the study; proportion male; mean age; diagnostic information; length of follow-up; mean IQ; proportion of participants with IQ ≥ 70 (in childhood and adulthood); outcome data (number of participants in each outcome category). DM and a third reviewer, SJC, independently completed the data capture form from each included study. Consensus was achieved by comparing data capture forms; any disagreement was resolved by discussion. Queries that could not be resolved were discussed with the remaining authors until consensus was achieved (all data discrepancies were resolved by DM and SJC).

### Risk of Bias

Risk of bias assessments in systematic reviews are essential as conclusions drawn from each included study need to be considered against the strengths and limitations of those studies (Siddaway et al. 2019). While outcome studies do not always present clear information about recruitment and study populations (which may hamper a systematic review of bias; Steinhausen et al. 2016) it is still important to consider other sources of methodological quality or bias for interpreting the literature and informing future studies. Consequently, this study used a modified version of the Downs and Black (1998) checklist for the assessment of methodological quality in healthcare interventions. The measure was modified, and tested, by both DM and SJC using one study included in the review. After completing the measure independently both DM and SJC discussed how applicable each item was to the studies under review. If an item was not appropriate for the included studies it was removed (e.g. one question was about randomisation of subjects and this was removed as it was deemed inappropriate for outcome studies). Six items were used to assess the quality of reporting of studies (are the aims and objectives clearly stated?; are the main outcomes to be measured clearly described?; is the sample clearly described?; are the main findings clearly described; are estimates or variability in the data described?; and have characteristics of patients lost to follow-up been clearly described?); items two and five were used to assess external and internal validity respectively (e.g. how representative is the sample, and were losses to follow-up taken into account respectively). DM and SJC independently rated each study. Siddaway et al. (2019) suggest that assessment tools should be used to reflect qualitatively on the strengths and limitations within and between studies, rather than commenting on quantitative scores. Thus, we also report qualitative findings from the quality and risk of bias assessments.

### Summary Measures

Due to the absence of, or small numbers in, some outcome ratings, outcome categories were collapsed as follows: very good and good outcomes were categorised as good; fair and restricted outcomes were categorised as fair; very poor and poor outcomes were categorised as poor. Most outcome studies included only autistic participants, but one study included a comparison group of participants with Down syndrome (Esbensen et al. 2010) and two compared autistic disorder with Asperger’s syndrome (Billstedt et al. 2005; Cederlund et al. 2008). The pooled proportion of outcome, with 95% confidence intervals (CI) was estimated for each of the three collapsed outcome categories.

### Analysis Plan

Meta-analysis was conducted using MetaXL (EpiGear 2016). Estimated prevalence, variance, and study weights were calculated for each outcome category under an Inverse Variance Heterogeneity model (IVhet; Doi et al. 2015). This model has been shown to produce more reliable parameter estimates compared to fixed-effect or random-effects meta analyses especially as between-study heterogeneity increases (Doi et al. 2015). As some studies recruited samples from the same cohort (e.g. Farley et al. 2009 and Farley et al. 2018) the most recent study from each cohort series was included, to avoid ‘double counting’ of participants. Two sub-group analyses (carried out in MetaXL) were used to compare: (i) studies with a mean age at follow-up of ≤ 29 years, or 30 + years; and (ii) samples containing participants diagnosed with childhood or infantile autism, or those with autism spectrum disorders (mixed samples were included in the latter category). We also conducted a post-hoc analysis based on the estimated year of baseline assessment; see Supplementary Material 3. (We thank an anonymous reviewer for this suggestion.) Cochran’s *Q*-statistic was used to assess the heterogeneity of the observed variation. The *Q*-statistic tests the null hypothesis that observed variation between effect sizes is due to chance (West et al. 2010). The extent of the heterogeneity was expressed using the *I*^2^ statistic, which expresses the percentage of the observed variance that is attributable to a real, and not chance, effect (Borenstein et al. 2017; West et al. 2010). Cut-offs for identifying low (25%), moderate (50%), and high (75%) heterogeneity have been proposed (Higgins et al. 2003). Finally, to account for multiple comparisons, a Bonferroni correction was applied (Abdi 2007) and a *p*-value of ≤ 0.01 was set for determining significance.

### Additional Analyses

Sensitivity analyses were conducted with the leave-one-out approach (van Heijst and Geurts 2015). Each study was sequentially removed from the analysis of each outcome category and each outcome proportion was re-estimated. A second sensitivity analysis was conducted by removing all studies that did not have information about diagnosis in *childhood*. For instance, Otsuka et al. (2017) report type of diagnosis (e.g. Asperger’s, PDD-NOS etc.), however, they do not indicate *when* their participants received their diagnoses. Thus, this analysis was included to see how robust the findings were when only studies with childhood diagnoses were included (as participants diagnosed later in life may differ from those diagnosed in childhood). Publication bias was not assessed for two reasons. First, for the meta-analysis the percentage in each outcome category *within* each study was estimated. As this requires three meta-analyses to be performed (for good, fair, and poor outcome) it would be difficult to interpret the three separate funnel plots. Second, follow-up studies are less likely to be affected by publication bias (Steinhausen et al. 2016). Findings of poor outcome would strengthen the consensus of existing studies; conversely, studies reporting positive outcomes would likely be published due to their novel findings.

### Meta-regression

The meta-regression was carried out using the ‘regress’ command in Stata 16 (StataCorp 2019). A multiple meta-regression model was used to identify study-level characteristics that predicted the proportion of each outcome category. The dependent variable was a percentage, which was converted to a proportion (with range 0–1), then a logit transformation was used to transform the proportion of each study-level outcome category (good, fair, poor). This creates a linear variable that can extend beyond the 0–1 range that defines a proportion, to prevent the variance of each study being ‘squeezed’ when proportions are close to 0 or 1 (Barendregt et al. 2013). The independent variables were study year, mean age of the sample, proportion of male participants, proportion of participants with an IQ greater than or equal to 70 in childhood and in adulthood. Note, we include study year as a continuous variable to investigate whether more recent studies are associated with better outcomes; this is distinct from the subgroup analysis defined above (which sought to distinguish outcomes based on diagnosis categories). Planned analyses were to regress transformed scores of each outcome category onto independent variables that significantly correlated with outcome scores. Regression coefficients were then exponentiated to facilitate interpretation.

## Results

### Study Selection

The search strategy returned a total of 8478 records. After removing duplicate records (N = 4209) the first author screened a total of 4339 records at the title and abstract level. GRS screened a random 10% of the records to assess reliability (agreement between DM and GRS was 98.5%). In line with recent recommendations (Siddaway et al. 2019), all the records on which there was disagreement (N = 7) were also included for a full-text read-through (N = 63; thus, the aim was to emphasise sensitivity over specificity at the screening stage). After completing the full-text read-through, 18 articles were included in the quantitative synthesis. There was a degree of overlap with this analysis and the previous meta-analysis conducted by Steinhausen and colleagues. This paper includes nine papers reported in the previous meta-analysis (1., 2., 3., 4., 5a., 5b., 6., 7., 8., and 9.; see Table 1 for study numbers), plus nine papers that were not included in the previous analysis (10., 11., 12., 13., 14., 15., 16., 17., and 18.). Three studies from the Steinhausen meta-analysis were excluded because the mean age of the participants was below the criterion set for this review (Rutter et al. 1967; DeMyer et al. 1973; Lotter 1974); a further three were excluded as a more recent study using the same cohort has since published (Farley et al. 2009; Howlin et al. 2004, 2013).

**Table 1:** Study characteristics for included studies

Study	Mean age	Year of baseline assessment^a^	N^b^	% male	Diagnostic type^c^	% with IQ ≥ 70 in childhood	% with IQ ≥ 70 in adulthood	Collapsed outcome categories		
								Good N (%)	Fair N (%)	Poor N (%)
1.Gilberg and Steffenberg (1987)^†^	19.8	–	23	73.9	IA	–	26.0	1 (4.3)	11 (47.8)	11 (47.8)
2. Kobayashi et al. (1992)^†^	21.8	1977	197	84.3	IA	23.6	–	53 (26.9)	53 (26.9)	91 (46.2)
3. Larsen and Mourisden (1997)^†^	37.8	1950	18	55.6	ASD	77.8	–	5 (27.8)	5 (27.8)	8 (44.4)
4. Engstrom et al. (2003)^†^	31.4	–	16	56.3	ASD	100	100.0	2 (12.5)	12 (75.0)	2 (12.5)
5a. Billstedt et al. (2005)^†^	25.5	1987	73	78.2	IA	20	7.0	0 (0.0)	18 (24.7)	55 (75.3)
5b. Billstedt et al. (2005)^†^	25.5	1987	35	54.8	ASD	14	3.0	0 (0.0)	5 (14.3)	30 (85.7)
6. Cederlund et al (2008)^†^	24.5	2001	70	43.0	IA	18	17.0	0 (0.0)	17 (24.3)	53 (75.7)
7. Eaves and Ho (2008)^†^	24.0	1986	47	77.1	ASD	17.4	–	10 (21.3)	15 (31.9)	22 (46.8)
8. Esbensen et al. (2010)^†^	37.7	–	70	65.7	ASD	–	0.0	7 (10.0)	20 (28.6)	43 (61.4)
9. Gillespie-Lynch et al. (2012)^†^	26.6	1989	20	100.0	IA	–	–	6 (30.0)	4 (20.0)	10 (50.0)
10. Chamak and Bonniau (2016)	18–54^d^	–	76	65.8	–	–	–	23 (30.3)	0 (0.0)	53 (69.7)
11. Moss et al. (2017)	47.9	1976	52	82.7	IA	100.0	–	5 (9.6)	15 (28.8)	32 (61.5)
12. Otsuka et al. (2017)	27.7	–	41	53.7	ASD	–	–	16 (39.0)	11 (26.8)	14 (34.1)
13. Helles et al. (2017)^e^	29.9	1992	50	100.0	ASD	100.0	100.0	18 (36.0)	29 (58)	3 (6.0)
14. Jonsdottir et al. (2018)	21.9	1996	15	86.7	IA	–	–	5 (33.3)	5 (33.3)	5 (33.3)
15. Farley et al. (2018)	35.5	1986	151	75.7	IA	–	23.0	30 (19.9)	52 (34.4)	69 (45.7)
16. Mason et al. (2019)	61.5	–	69	69.6	ASD	–	–	11 (20.4)	31 (57.4)	12 (22.2)
17. Sevaslidou et al. (2019)	19.4	1998	53	83.0	–	–	100.0	22 (41.5)	6 (11.3)	25 (47.2)
18. Pickles et al. (2020)^e^	26.1	1997	123	86.7	ASD	–	50.6	48 (47.5)	16 (15.8)	37 (36.6)

‘–’ data not available

^a^Estimated from data provided in each paper; where data were collected over multiple years, the median year in the interval was calculated

^b^Total N does not always equal total of outcome scores due to missing data, or deceased participants

^c^Infantile/Childhood Autism (IA) or Autism Spectrum Disorders (inc. Asperger’s, PDD-NOS etc.; ASD)

^d^Mean age not reported

^e^Data provided by personal communication; † denotes studies that were included in the previous meta-analysis carried out by Steinhausen et al. (2016)

### Study Characteristics

Table 1 presents the study characteristics relevant to the present analyses. The 18 studies were based on a total of 1199 autistic participants (19 samples are included as Billstedt et al. 2005 reported on two separate autistic cohorts). Year of publication ranged from 1987 to 2020. Mean age of the participant groups was mostly below 50 years, with one exception (Mason et al. 2019; mean age = 61.5). The proportion of male participants ranged from around 50% to 100%. The proportion of participants with an IQ ≥ 70 ranged from around 20% to 100% for childhood IQ and 0% to 100% for adult IQ. Eight studies (nos. 1., 2., 5a., 6., 9., 11., 14., and 15.) included participants diagnosed with infantile or childhood autism; nine (nos 3., 4., 5b., 7., 8., 12., 13., 16., and 18.) reported on samples of participants diagnosed with autism spectrum disorder (two, nos 10., and 17., provided insufficient information on diagnosis). Although all studies reported numbers of males and females, neither IQ nor outcome scores were stratified by gender (except study 17.).

### Risk of Bias

Agreement between DM and SJC was high (Cohen’s κ = 0.839, *p* < 0.001) for both methodological quality and risk of bias assessments. All studies reported the relevant outcomes in line with either the Howlin et al. (2004) or Lotter (1974) criteria. Many made direct reference to these criteria, or in the case of some studies expressed the outcome as levels of independence, scored from very high to very low or very independent to very limited (Esbensen et al. 2010 and Farley et al. 2018 respectively). However, no studies, apart from Farley et al. (2018), explicitly described a process of reliability checking for the coding of outcomes. Representativeness of samples was the main risk of bias across most studies. Eight of the samples recruited their participants from hospital or therapeutic settings (nos 2., 7., 9., 10., 11., 12., 14., and 17). Several studies attempted to recruit a more representative sample of participants from the population from national data registries, community samples, or epidemiological studies of autism prevalence (nos 1., 4., 5a., 5b., 6., 13., and 15.). Three studies did attempt to assess if responders differed from non-responders at the most recent follow-up (nos 9., 13., and 15.), with all three reporting that responders tended to have significantly higher IQ at previous times points than non-responders (e.g. Farley et al. 2018 reported an average difference of 9 points).

### Synthesis of Results

The pooled estimate for the percentage of participants with a good outcome was 20.0% (95% CI 10.9–30.1); for fair outcome the percentage was 26.6% (95% CI 17.5–36.2), and for poor outcome the percentage was 49.3% (95% CI 38.1–60.5). Note, due to the logit transformation and subsequent back transformation, proportions do not always sum to 100% when there are three or more categories (Barendregt et al. 2013). Q-values ranged from 158.9 to 204.5 and were significant for each outcome category (all *p* < 0.001) indicating significant heterogeneity between studies not due to chance or sampling error. The *I*^2^ values ranged from 88.7% to 91.2% indicating that heterogeneity between studies was high for each outcome category (Borenstein et al. 2017). See Table 2 for the estimated proportions for each outcome category (with CIs), heterogeneity statistics, and *p*-values.

**Table 2:** Pooled outcome proportions, with CIs, and heterogeneity statistics

Outcome category	Estimated proportion (%)	CI	*Q*-statistic	*p*-value	*I*^2^ (%)
Good	20.0	[10.9–20.1]	204.5	< 0.001	91.2
Fair	26.6	[17.5–36.2]	158.9	< 0.001	88.7
Poor	49.3	[38.1–60.5]	178.5	< 0.001	89.9

### Subgroup Analyses

Due to significant, and large, heterogeneity between studies, sub-group analyses were conducted in line with the previous meta-analysis (Steinhausen et al. 2016). A Z-test of two proportions was used to assess whether the differences in outcome proportions significantly differed between subgroups (Ware et al. 2012). Age at follow-up was significantly related to fair outcome proportion. Younger participants (mean age $\le$ 29 years) were significantly less likely to be in the fair outcome group compared with those aged ≥30 years (22.1% vs. 37.4%; *Z* = 5.64, *p* < 0.001). The proportions of those in the good and poor outcome categories did not differ for those above or below 30 years at follow-up (*Z* = 1.99, *p* = 0.047 and *Z* = 1.72, *p* = 0.086 respectively; see Table 3). The impact of diagnostic category was also explored; individuals diagnosed with childhood/infantile autism were compared with those who had a diagnosis of autism spectrum disorders. Diagnosis was significantly related to both the good and poor outcome categories. The proportion of participants in the good category was significantly lower for those diagnosed with childhood/infantile autism compared to other autism spectrum disorders (17.4% vs. 26.0%; *Z* = 3.42, *p* < 0.001). Individuals with a diagnosis of autism spectrum disorder were significantly less likely to be rated as having a poor outcome than those diagnosed with infantile/childhood autism (41.2% vs. 53.2%, *Z* = 3.83, *p* < 0.001). See Table 3 for the summary statistics for the sub-group analyses. The difference for the fair outcome category was non-significant (25.3% for infantile autism vs. 29.4% for autism spectrum disorders, *Z* = 1.48, *p* < 0.139). When considering estimated year of baseline assessment, good outcomes were significantly higher for those assessed in childhood more recently (i.e. after the median year, 27.8% compared to those assessed before the median year, 17.5%; Z = 3.646, *p* < 0.001); conversely poor outcomes were significantly lower for those assessed more recently (42.0% for those assessed after the median year compared to 51.9% for those assessed before the median year; Z = 2.869, *p* < 0.01). There was no difference in the fair outcome category (Z = 1.020, *p* = 0.308).

**Table 3:** Sub-group analyses by age at follow-up and diagnosis type

	Pooled outcome proportion [CIs]		Pooled outcome proportion [CIs]	
	≤ 29	30 +	IA group*	ASD group*
Good	21.6% [8.1–36.9]	20.0% [10.9–30.1]	17.4 [6.4–30.1]	26.0% [10.8–42.8]
Fair	22.1% [11.9–33.3]	37.4% [24.1–51.1]	25.3% [13.0–38.6]	29.4% [16.4–43.3]
Poor	50.9% [35.9–65.8]	45.7% [28.8–62.9]	53.2% [41.3–65.0]	41.2% [21.0–62.2]

### Additional Analyses

Sensitivity analyses indicated that the meta-analysis models were robust for each outcome category when each study was removed. Estimated proportions for good, fair, and poor outcomes ranged from 17.8% to 22.3%, 25.2% to 29.6%, and 47.5% to 51.6%, respectively. When studies with no clear indication of a diagnosis of ASD in childhood were excluded, the pattern of results was largely unchanged, with estimated proportions of good outcome = 19.3% [CI = 7.6–32.7%]; fair outcome = 27.5% [CI = 20.8–34.6%]; and poor outcome = 49.7% [CI = 36.5–62.8%].

### Meta Regression

It was not possible to extract sufficient data on language and autism severity from the included papers; thus IQ, study year, mean sample age, and proportion of males were investigated as predictors of outcome. Study-year, mean sample age, and proportion of males did not correlate with any of the transformed outcome scores (all *p* > 0.01). IQ in childhood significantly correlated with fair outcome proportion (r = 0.706, *p* < 0.01) and poor outcome proportion (r = − 0.715, *p* < 0.001) indicating higher IQ was associated with a greater proportion of adults in the fair, and a lower proportion of adults in the poor, outcome categories. IQ in adulthood correlated significantly with good outcome (r = 0.650, *p* < 0.001), and negatively with poor outcome (r = − 0.876, *p* < 0.001). IQ in childhood and IQ in adulthood were highly correlated (r = 0.996, *p* < 0.05). As some studies only reported IQ in childhood or in adulthood, and combining IQ data from these two developmental ages seemed inappropriate, it was decided that separate models for adulthood and childhood should be estimated. See Table 4 for all correlation co-efficients. Thus, the following regression models were computed: good and poor transformed outcome scores were regressed (separately) onto IQ in adulthood; fair and poor transformed outcome scores regressed onto IQ in childhood (separately). Table 5 shows the results of the regression analyses. Lower IQ in adulthood significantly predicted poor outcome, explaining 73.8% of the variance (β = − 0.493, *p* = 0.01), but not the proportion of good outcomes (variance explained = 35.0%, β = 0.512, *p* = 0.042). Despite significant correlations, IQ in childhood did not significantly predict variance in the fair or poor outcome proportions at the adjusted alpha level (variance explained = 42.7%, β = 0.503, *p* = 0.034 and variance explained = 44.1%, β = − 0.494, *p* = 0.031 respectively).

**Table 4:** Correlations between selected predictors and transformed effect size for outcome

	Transformed effect size		
	Good	Fair	Poor
Study year	0.323	− 0.203	− 0.191
Sample age	0.137	0.377	− 0.192
Proportion male	0.457	0.076	− 0.349
Childhood IQ	0.602	0.706***	− 0.715***
Adulthood IQ	0.650***	0.599	− 0.876***

**Table 5:** Variance explained from each meta-regression model

	Adjusted R^2^	Predictor	β (exponentiated)	*p*-value
Good	0.350	IQ in adulthood	0.512	0.042
Fair	0.427	IQ in childhood	0.503	0.034
Poor	0.441	IQ in childhood	− 0.494	0.031
	0.738	IQ in adulthood	− 0.493	0.001

## Discussion

This analysis included 1,199 participants, with a wide range of diagnosis types (e.g. autistic disorder, Asperger’s syndrome, PDD-NOS) and nationalities (e.g. the United Kingdom, United States of America, Japan, and Sweden). The proportion of participants with an IQ above 70 was highly variable across studies (from 0 to 100%) and the proportion of males varied from approximately 50–100%. The pooled proportions of participants rated as having good, fair, and poor outcomes were estimated to be approximately 18%, 28%, and 50% respectively. This is the first analysis of outcome data in autism to use a meta-regression approach to identify study-level predictors of outcome. We identified a link between IQ measures in childhood and adulthood that were predictive of outcome. The present findings will be discussed in line with previous reviews below.

The estimated outcome percentages in the present study largely agree with the previous meta-analysis of the same type of outcome studies. Thus, it does seem to be safe to conclude that approximately 50% of autistic people will struggle to achieve, and only around 20% *will* achieve, traditional markers of objective functioning (i.e. being competitively employed, having friends or romantic relationships, and living independently). This finding is striking, given this study included nine new studies not previously reported on. Thus, despite five years and nine new outcome studies, it appears autistic people still struggle to be independent (as conceptualised in this review). This study highlighted that IQ in childhood was a predictor of fair and poor outcomes. This is consistent with reviews conducted by Kirby et al. (2016) and Magiati et al. (2014). Both reviews qualitatively report that IQ in childhood is a predictor of outcome; moreover, Kirby et al. note that age is not a predictor of outcome. Thus, this review formally analysed these associations and is consistent with the previous reviews. What is important to note, is the utility of repeating these analyses periodically. Farley et al. (2009)’s paper is highlighted in the Magiati et al. (2014) review as an example of relatively better outcomes compared to other studies (with 48% having a very good or good outcome, and 34% a fair outcome). However, at a subsequent follow-up the very good and good outcome proportions were less than half (20%). In sum then, this review is broadly consistent with previous findings and adds a quantitative assessment of the role of IQ at the study level in predicting outcomes; until now this had only been qualitatively described.

To account for the high between-study heterogeneity we undertook two subgroup analyses based on age at follow-up and on diagnostic category. In the first analysis, the proportion of fair outcomes differed significantly, with more adults aged 30+ in this category, compared to fair outcomes in those aged ≤ 29 years (good and poor outcomes did not differ). Our results are inconsistent with Steinhausen et al. (2016), who report significant differences for good and poor outcomes, but not for fair outcomes. This is likely explained by the categories examined. Steinhausen and colleagues compared three age groups (< 20 years, 20–29 years, and 30 + years); due to our exclusion criteria, we could only compare those samples followed up at a mean age of 29 years or less, versus those aged 30 years or more. The second subgroup analysis showed that those diagnosed with infantile/childhood autism were less likely to have a good outcome, and more likely to have a poor outcome compared to those diagnosed with autism spectrum disorders. This is somewhat consistent with Steinhausen et al. (2016) who found the same association for poor outcome, however, Steinhausen and colleagues found an association between fair outcome but not good outcome. This may be explained by the inclusion of two very recent studies (Pickles et al. 2020; Sevaslidou et al. 2019) which reported good outcomes in around 40%; thus, these studies likely alter the pattern of association due to their higher proportions of good outcome. As an alternative analysis we examined a median split based on an estimate of the baseline year of childhood assessment (see Supplementary Material 3). This did reveal that good outcomes were significantly greater, and poor outcomes significantly lower, for those assessed in childhood or more recently (i.e. after the median year). Yet, an inspection of Table 1 shows both diagnostic categories (infantile/childhood autism and autism spectrum disorder) appear before and after the median year. This suggests these diagnostic categories (infantile/childhood autism and autism spectrum disorders) are not a useful comparison; moreover, these categories do not necessarily conform to current diagnostic criteria (Constantino and Charman 2016) which in turn may artificially create differences based on inaccurate categorisation of participants.

While higher IQ has often been associated with a more positive outcome (Eaves and Ho 2008; Howlin et al. 2004, 2013; Rutter et al. 1967) findings on the predictive value of early symptom severity *combined with* IQ are often inconsistent (i.e. no relationship to outcome, e.g. Eaves and Ho 2008; or severity as a stronger predictor than IQ, e.g. Howlin et al. 2013). To address these mixed findings a meta-regression analysis was conducted: IQ in childhood and IQ in adulthood were used to predict outcome proportion (year of study, mean age, and proportion male were not related to outcome proportion). Only IQ in adulthood was a significant predictor of *poor* outcome proportion (IQ in adulthood did not predict good or fair outcome and IQ in childhood did not predict fair or poor outcomes when controlling for multiple comparisons); increased proportion of participants with IQ ≥ 70 predicted lower proportion of participants with a poor outcome. However, the regression *p*-values and β weights were included for all tested regression models for three reasons. First, as multiple comparisons can increase type II error rate (Rothman 1990), we present these findings to allow the reader to weigh up the effect sizes in light of the other statistical tests. Second, because the variance explained was reasonably high (around 50%) for each model, given the relatively small number of studies, effect sizes may be more informative when results are non-significant (Althouse 2016). Third, the small number of studies and predictors may mean the models lack statistical power (not that the effect of IQ is non-significant). Thus, these results should be interpreted very cautiously. However, given changes in cognitive functioning over time, it seems plausible that IQ in adulthood is likely to be more closely related to adult outcome than childhood IQ (with higher IQ being associated with a higher proportion of good, and fair outcomes, and a lower proportion of poor outcome). Future studies should seek to clarify this relationship by examining other factors associated with IQ which may also influence outcome. For instance, autistic adults with IQ in the average range may be able to obtain employment, but may struggle to keep it (Taylor et al. 2015) and Anderson K.A. et al. (2014) found that autistic adults without intellectual disability were less likely to live independently or to have obtained employment compared to non-autistic adults with intellectual disability. Social factors, such as attendance in mainstream schooling may also exert an influence on outcome, potentially more so than IQ (Simonoff et al. 2019). These studies suggest that behavioural, cognitive, or social factors not necessarily associated with IQ may affect outcome. Moreover, psychiatric conditions may be associated with outcome. Sevaslidou et al. (2019) reported that presence of a co-occurring condition was associated with poorer outcome and Esbensen et al. (2010) reported that receiving psychiatric services was associated with poorer outcome. Therefore, while it is important for future studies to continue reporting cognitive ability, IQ alone should no longer be a primary focus for predicting outcome.

The main finding from the methodological assessment was that participants included in these outcome studies are unlikely to be representative of the autism population as a whole. Although many of the studies were longitudinal and reported on the same, or similar, cohorts over several time points, these studies were often drawn from clinical or therapeutic settings (e.g. Kobayashi et al. 1992; Moss et al. 2017; Sevaslidou et al. 2019). Thus, inferences from this analysis could be confounded by potential systematic differences between the wider population and clinical samples. Similarly, in cross-sectional studies (Mason et al. 2019; Otsuka et al. 2017), it is unclear how representative these samples are of their respective populations.

Attrition is a further source of potential bias. Three studies reported that individuals lost to follow-up had significantly lower IQ’s at earlier time points, suggesting that cognitively able participants are more likely to take part in subsequent follow-ups (e.g. Helles et al. 2017). This has a clear implication for our understanding of outcomes in autism—autistic people of lower IQ are less likely to be represented as cohorts age. Difficulties for researchers in gaining access to sheltered or supported accommodation may also be partly responsible for the loss of less cognitively able participants (Farley et al. 2018).

It is also crucial to reflect on the concept of outcome itself. As noted in the introduction, definitions of outcome have shifted from subjective appraisals (based mainly on researchers’ own concepts) of how well the individual is doing, to more objective criteria measuring employment, independent living, and friendships/relationships. It is important to note that employment and independent living are directly observable in accordance with predefined criteria; there is a subjective element to assessing social relationships (as can be seen from the criteria in Supplementary Material 2). Although these latter criteria are more methodologically robust and replicable, such concepts of outcome are both restricted and reductionist and tend to assume (at least implicitly) that the source of the poor outcome lies within the individual (e.g. autism characteristics or IQ). However, since the late 1990s there have been attempts to redefine what constitutes ‘success’ for autistic people (Georgiades and Kasari 2018; Henninger and Taylor 2013; Lounds Taylor 2017; Ruble and Dalrymple 1996). Ruble and Dalrymple (1996) put forth a compelling argument that many aspects of success are lost when only objective criteria of outcome are used. Yet, they also recognise that outcome should be measured, in part, as a set of risk and protective factors. Therefore, employment may be a protective factor, as it provides an income (which in turn facilitates a level of independence), or unemployment may be a risk factor (leading to financial dependence on others). Nevertheless, Ruble and Dalrymple (1996) contend that success can be conceptualised as something *more than* the state of employment. Both Lounds Taylor (2017) and Georgiades and Kasari (2018) suggest that outcome should be measured relative to the individual. For instance, employment itself is only one aspect of outcome; how individuals fare over the course of their lives is also critical. Thus, an autistic person who has low educational attainments, but then pursues a hobby or interest and has positive social interactions with the community can be conceptualised as having a good outcome (examples of such alternative accounts of outcome can be found in Ruble and Dalrymple 1996). Recently, some studies have measured both traditional objective measures of outcome and some aspects of personal subjective experience (e.g. sense of coherence; Helles et al. 2017), but as yet there is no robust framework for integrating these objective and subjective domains. Hence, although traditional measures of outcome are important, for example for comparisons with other groups of individuals (Shattuck et al. 2020), consideration of each individual’s perspective is vital in helping to define what “success” really means. Following from this, future research should seek to integrate the autistic person’s valued “beings” and “doings” (thought to be central to some objective-subjective theories of quality of life; e.g. Ruta et al. 2007); this will help move conceptualisations away from deficit (or deficit only) models, towards models that recognise each individual’s strengths, as well as their challenges (Robertson 2009; Ruble and Dalrymple 1996).

### Strengths and Limitations

The present meta-analysis has several strengths. This review used a meta-analytical model that has recently been shown to be more robust to high inter-study heterogeneity compared with other models (hence providing more conservative estimates and confidence intervals; Doi et al. 2015). Consequently, these results, plus those of the previous meta-analysis (Steinhausen et al. 2016), indicate a consensus about the estimated proportions of autistic adults who have poor, fair or good outcomes. Our meta-regression models of IQ and outcome represent a novel and important contribution to the literature (see Pickles et al. 2020 for a latent profile analysis that draws a similar conclusion). Whilst most of these models were non-significant at the adjusted *p*-value, the results were presented due to considerations about statistical power and potential type II errors. As outlined in the introduction, our protocol used two independent reviewers for screening (GRS) and data extraction (SJC; reliability was high for both processes). Third, this study undertook additional analyses that extend the findings of the previous meta-analysis. A sensitivity analysis was performed to gauge how robust the findings are, which indicated that estimates were not being inflated by an ‘outlier’ study.

Limitations of the present meta-analysis must also be acknowledged. The main issue affecting the interpretation of data is the measurement of outcome. Moreover, as interpretations of the criteria used vary across studies this confounds between study comparisons. Therefore, although the pattern of outcomes (few good, and many poor, outcomes) is broadly similar across studies it is not feasible to assess how measurement differences affect the conclusions. Second, we replicated the subgroup analysis conducted by Steinhausen and colleagues. However, as noted above, this analysis is problematic, given the lack of precision with which diagnostic categories are applied. Thus, although we found some similar results it is unclear how meaningful this analysis is at anything but a broad conceptual level. It was necessary to estimate a time point to compare those assessed in childhood across time (i.e. those assessed in childhood decades ago, versus those assessed in childhood more recently). We estimated a median year of assessment based on the information presented in each paper (where possible). However, it must be acknowledged this is a crude index of assessment, and was not possible for all papers. Thus, if such information was available for all studies the pattern of results may be different. A third potential limitation is the very high between-study heterogeneity, with each outcome category showing heterogeneity greater than 88%. Therefore, it could be argued that these studies are too heterogeneous to compare using a meta-analysis. However, the meta-analytical model used has been shown to be robust to high levels of between study heterogeneity. Moreover, those regression models that were significant explained just over 50% of the variance. Thus, the association with IQ does help to account for some between-study differences. It is likely that there are many unexplored factors contributing to this heterogeneity (e.g. participant level characteristics, such as decision-making style; or contextual factors, such as socio-economic status that have yet to be measured in outcome studies); as the number of published outcome studies increases future meta-analyses will be able to investigate these and other variables in more detail. Thinking prospectively about future analyses of outcome data is important; the number of studies in this review is small, thereby reducing the statistical power to detect effects. Moreover, other variables that have been hypothesised to be predictive of outcome (e.g. autism severity and language) were not factored into the present analysis as sufficient data for analysis were often unavailable. Thus, any future meta-regression should assess the contribution of other theoretically important variables in predicting outcome. Finally, the use of a standardised checklist is recommended to assess studies’ quality and risk of bias (Siddaway et al. 2019). However, since many such measures exist (Siddaway et al. 2019 report the number to be 86), a future review using a different measure may find subtly different methodological problems/strengths. This is problematic for comparing quality/risk assessments across reviews. Indeed, varied measures of a range of domains (e.g. autism symptoms, quality of life, and cognition/language) is common in autism research (Magiati et al. 2014). It may be that researchers should attempt to identify paradigmatic measures that are consistently used to facilitate cross-study comparisons (Roestorf et al. 2019).

## Conclusion

Social outcomes for the majority of autistic adults are poor; approximately 50% fail to achieve independence in their living status, employment, and close relationships. These findings broadly agree with the previous meta-analysis of outcome by Steinhausen et al. Nevertheless, heterogeneity of the findings for each outcome category was high. This suggests that the proportion of each outcome is influenced by systematic factors and not sampling error. Finally, a series of meta-regression models was carried out to investigate heterogeneity between studies. IQ in adulthood significantly predicted the proportion of individuals rated as having a poor outcome, but neither IQ in childhood or adulthood significantly predicted proportions of good and fair outcomes when adjusting for multiple comparisons. Based on a robust methodological assessment future studies should accurately characterise participant samples based on theoretically important variables and future studies should try to account for the representativeness of their samples. Finally, the concept of ‘outcome’ assessed in these studies is highly reductive; it assumes intra-individual factors drive outcome, to date very few studies have looked at how the broader familial and contextual status of autistic people is related to outcome.

## Electronic supplementary material

Below is the link to the electronic supplementary material.

Supplementary file1 (DOCX 13 kb)

Supplementary file2 (DOCX 16 kb)

Supplementary file3 (DOCX 13 kb)

## Footnotes

1 ASD will be used as this is a diagnostic term (e.g. DSM-5) and is used throughout the literature. However, when referring to participants within studies ‘autistic person’ will be used to better reflect the neurodivergent position that autism need not be characterised as a disorder.
