# Supplementary material for: A Meta-analysis of Outcome Studies of Autistic Adults: Quantifying Effect Size, Quality, and Meta-regression
Source: J Autism Dev Disord. 2020 Nov 17;51(9):3165–79. doi: 10.1007/s10803-020-04763-2 (PMC8349337; doi:10.1007/s10803-020-04763-2)
Supplement: Supplementary file 1 — Supplementary file1 (DOCX 13 kb) [file 10803_2020_4763_MOESM1_ESM.docx]

| 1 | exp Autism Spectrum Disorders/ |
| --- | --- |
| 2 | autis*.tw. |
| 3 | asperger*.tw. |
| 4 | adult*.tw. |
| 5 | adolescen*.tw. |
| 6 | teenag*.tw. |
| 7 | outcome*.tw. |
| 8 | exp Longitudinal Studies/ |
| 9 | longitudinal*.tw. |
| 10 | traject*.tw. |
| 11 | prognos*.tw. |
| 12 | exp Prediction/ |
| 13 | predict*.tw. |
| 14 | 1 or 2 or 3 |
| 15 | 4 or 5 or 6 |
| 16 | 7 or 8 or 9 or 10 or 11 or 12 or 13 |
| 17 | 14 and 15 and 16 |
| 18 | limit 17 to yr="2000 -Current" |

Supplementary material 1 – Search strategy for PsychINFO database
