# Supplementary material for: A Meta-analysis of Outcome Studies of Autistic Adults: Quantifying Effect Size, Quality, and Meta-regression
Source: J Autism Dev Disord. 2020 Nov 17;51(9):3165–79. doi: 10.1007/s10803-020-04763-2 (PMC8349337; doi:10.1007/s10803-020-04763-2)
Supplement: Supplementary file 2 — Supplementary file2 (DOCX 16 kb) [file 10803_2020_4763_MOESM2_ESM.docx]

Supplementary material 2 – Definitions of outcome

Rutter/Howlin criteria (from Howlin et al., 2004; 2013):

| Quantitative score | Outcome category | Definition |
| --- | --- | --- |
| 0-2 | Very good | A high level of independence: including some friends and a job |
| 3-4 | Good | In work, but requires some daily living support; some friends/acquaintances |
| 5-7 | Fair | Some independence, requires support and supervision but does not need residential support. No close friends, but some acquaintances |
| 8-10 | Poor | Specialist residential support needed or high level of support across domains, no friends outside of residence |
| 11 | Very poor | High-level hospital care, no friends, no autonomy |

Each of the three outcome domains was assessed using the following criteria:

*Occupation*:

- 0, employed, or self-employed
- 1, volunteer work, job training or low-pay scheme
- 2, supported employment or sheltered employment
- 3, special centre/no occupation

*Friendship*:

‘Frequency’ was rated based on number of friends/acquaintances; ‘Quality’ was based on the degree of sharing/mutual participation.

- 0, one or more close friendships of good quality (mutual sharing of confidences and a range of activities undertaken together)
- 1, one or more friends but of poorer quality and a restricted range of interests undertaken together
- 2, no specific friendships, but the individual seeks contact with others in group situations
- 3, no friends, no joint activities

*Independence*:

- 0, living independently
- 1, semi-sheltered accommodation, or still at home with a high degree of autonomy
- 2, living with parents, but with limited autonomy
- 3, residential accommodation with some autonomy
- 4, specialist autistic or other residential accommodation; little or no autonomy
- 5, hospital care, or at home (because no alternative accommodation could be found)

Alternative criteria (e.g. Billstedt et al., 2005):

*Good*: (a) being employed/in higher education/vocational training AND (b) living independently (if aged 23 years of older OR having two or more friends/a steady relationship (if aged 22 years or younger)

*Fair*: either (a) OR (b) as outlined above

*Restricted*: neither (a) NOR (b) as outlined above AND not meeting criteria for a major psychiatric disorder. This category is for those who meet the poor outcome criteria BUT have been accepted by a peer/social group such that “their handicaps are not so readily obvious”

*Poor*: “severe handicap”, no independent social progress but some verbal or non-verbal communication skills

*Very poor*: “very severe handicap”, unable to lead an independent life, no clear verbal or non-verbal communication
