# Supplementary material for: A Meta-analysis of Outcome Studies of Autistic Adults: Quantifying Effect Size, Quality, and Meta-regression
Source: J Autism Dev Disord. 2020 Nov 17;51(9):3165–79. doi: 10.1007/s10803-020-04763-2 (PMC8349337; doi:10.1007/s10803-020-04763-2)
Supplement: Supplementary file 3 — Supplementary file3 (DOCX 13 kb) [file 10803_2020_4763_MOESM3_ESM.docx]

Supplementary Material 3: Comparisons based on estimated year of baseline assessment

Not many papers reported the exact years during which baseline assessments were carried out. So, to facilitate this analysis we used the date of publication and any information in the paper about the mean age of assessment in childhood. It was not possible to estimate this for some studies (because the study was cross-sectional, or there was not adequate information in the paper).

We then analysed differences in outcomes based on those studies above, and below, the median year of baseline assessment. (Median = 1988)

| Supplementary table: Outcome scores based on median split of estimated baseline year of assessment | | | |
| --- | --- | --- | --- |
| Outcome category: | Before median year | After median  year | Z-test of two proportions  Z score; *p*-value |
| Good | 17.5% | 27.8% | 3.646; <0.001 |
| Fair | 27.4% | 24.3% | 1.020; 0.308 |
| Poor | 51.9% | 42.0% | 2.869; 0.004 |
